# Emerging Trends in the Formation and Function of Tuberculosis Granulomas

**DOI:** 10.3389/fimmu.2012.00405

**Published:** 2013-01-07

**Authors:** Geanncarlo Lugo-Villarino, D. Hudrisier, A. Benard, Olivier Neyrolles

**Affiliations:** ^1^CNRS, Institut de Pharmacologie et de Biologie StructuraleToulouse, France; ^2^Institut de Pharmacologie et de Biologie Structurale, Université de Toulouse, Université Paul SabatierToulouse, France

**Keywords:** macrophage, B-cells, mycobacteria, tuberculosis, granuloma

## Abstract

The granuloma is an elaborated aggregate of immune cells found in non-infectious as well as infectious diseases. It is a hallmark of tuberculosis (TB). Predominantly thought as a host-driven strategy to constrain the bacilli and prevent dissemination, recent discoveries indicate the granuloma can also be modulated into an efficient tool to promote microbial pathogenesis. The aim of future studies will certainly focus on better characterization of the mechanisms driving the modulation of the granuloma functions. Here, we provide unique perspectives from both the innate and adaptive immune system in the formation and the role of the TB granuloma. As macrophages (Mϕs) comprise the bulk of granulomas, we highlight the emerging concept of Mϕ polarization and its potential impact in the microbicide response, and other activities, that may ultimately shape the fate of granulomas. Alternatively, we shed light on the ability of B-cells to influence inflammatory status within the granuloma.

## Introduction

“On the basis of my numerous observations I consider it established that, in all tuberculous affections of man and animals, there occur constantly those bacilli which I have designated tubercle bacilli and which are distinguishable from all other microorganisms by characteristic properties.”

With those celebrated words in 1882, Koch announced the discovery of the etiological agent of one of the oldest recorded human afflictions (Koch, 1982). The term “tubercle” refers to an original description by Sylvius (in 1650) of the apparent lung nodules characteristic of the “consumption” disease, which became christened as “tuberculosis (TB)” by Schonlein (in 1839) in recognition of its intricate correlation with these structures (Sakula, 1982). Today, these tubercles are known as granulomas, defined as organized immune cell aggregates that form in response to persistent TB infection (Ramakrishnan, 2012). The cellular composition of TB granulomas includes Mϕs, neutrophils, monocytes, dendritic cells, B- and T-cells, fibroblasts, and epithelial cells (Russell, 2007; Ramakrishnan, 2012). Moreover, TB granulomas are characterized by a high-turnover rate of their Mϕ population and by specialized differentiations taking place in mature Mϕs such as tightly interdigitated cell membranes that make Mϕs appear either epithelial (Adams, 1974), fusion into multinucleated giant cells (Helming and Gordon, 2007), or differentiation into foamy cells with a high lipid content (Russell et al., 2009). While granulomas have been studied for about 200 years, their role in TB etiology remains unclear. In 1819, Laënnec first proposed granulomas as the cause of TB (Sakula, 1982). Yet, about a century went by before Ghon correlated the presence of a single caseous granuloma in the mid-region of the lung with a corresponding nodal involvement (the Ghon complex) and the pathogen’s dissemination, thus serving as a marker for latent TB (Dorhoi et al., 2011). In spite of this, subsequent studies and clinical observations established the granuloma as a host-protective structure that “walls off” Mtb to prevent its dissemination, a notion that still predominates. Seminal studies by Ramakrishnan in zebrafish, however, have now evidenced mycobacteria actually exploit the granuloma into a tool for pathogenesis, suggesting its function can be modulated depending on the disease context (Ramakrishnan, 2012). Considering TB is still one of the leading causes of human death due to a single infectious agent, substantial insights into microbe physiology and host defenses rest in the attempt to better understand the mechanisms governing TB granulomas.

Here, we will focus exclusively in the role of Mϕ polarization in the formation and function of TB granulomas. Likewise, we will provide a unique perspective on the significance of B-cells, whose immune-modulatory function has long been ignored in TB.

## Macrophage Polarization in TB Granulomas

Mϕ polarization is broadly classified into M1 and M2 programs (Goerdt and Orfanos, 1999; Gordon, 2003; Mantovani et al., 2004; Martinez et al., 2009). On one hand, the M1 program is a response to type-1 inflammatory conditions (e.g., IFN-γ), often associated with intracellular pathogen resistance (Quintana-Murci et al., 2007; Benoit et al., 2008). IFN-γ is mainly responsible for the establishment of the M1 program, granting Mϕs the capacity to kill mycobacteria (Flynn et al., 1993; Ehrt et al., 2001). The production of nitric oxide (NO) in Mϕs (characteristic in murine models) is arguably one of the most important consequences mediated by IFN-γ, as mice deficient for NO production succumb to Mtb infection (Chan et al., 1992). In fact, the enzyme iNOS (inducible NO synthase) required for NO production is a bona fide marker of murine M1 Mϕs (Xie and Nathan, 1993). Other marker genes, whose expression is induced in M1, include *ido1, ptgs2, il12b/il23a, socs3, marco, cd86, irf3/irf5*, and *stat1/stat5*, among others (Lawrence and Natoli, 2011; Murray and Wynn, 2011b). Collectively, the M1 program is part of the “common host response” against intracellular bacteria that endows Mϕs with a non-permissible nature (Ehrt et al., 2001; Deretic et al., 2004; Martinez et al., 2009; Cairo et al., 2011; Murray and Wynn, 2011a). On the other hand, the M2 program is dictated by type-2 inflammatory signals (e.g., IL-4, IL-10), enabling Mϕs to participate in the suppression of inflammation, phagocytosis, tissue remodeling, and repair, among others (Sica et al., 2008; Martinez et al., 2009; Murray and Wynn, 2011a). However, this program also renders Mϕs poorly microbicidal against intracellular pathogens (Raju et al., 2008; Martinez et al., 2009). This is best illustrated by how the arginine metabolism is used in M2 Mϕs, which shuts down NO production in favor of tissue reparation (Shearer et al., 1997). Indeed, M2 polarization is accompanied by ARG1 (type-1 arginase) expression that inhibits NO production by outcompeting iNOS to convert arginine into ornithine and urea (Munder et al., 1998; El Kasmi et al., 2008). Along arg1, other M2 marker genes include *fizz1, chi311/chi312/chi313, mrc1, cd36, socs2, il-10, klf4, jmjd3/irf4, pparγ*, and *stat6*, among others (Lawrence and Natoli, 2011; Murray and Wynn, 2011b). Altogether, Mtb might influence the granuloma function by controlling Mϕ polarization, a premise that is presciently in line with the following findings, which for the purpose of conciseness, are mainly based on the use of the iNOS/ARG1 polarization axis.

The animal models to study TB granulomas are discussed in detail elsewhere (Flynn, 2006). Here, we highlight recent findings in mice and zebrafish documenting the TB granuloma dynamics, supported by studies and clinical observations done in TB patients. It is widely postulated the onset of human pulmonary TB begins when inhaled Mtb is captured by Mϕs and transported across the alveolar epithelium into the lung tissue. In zebrafish, the subsequent steps leading to a nascent granuloma have been captured in real-time imaging (Davis et al., 2002). While infected Mϕs undergo apoptosis, they promote the recruitment of phagocytes, which upon arrival, display high motility conducive for scavenging apoptotic cells. The phagocytosis of dead Mϕs leads to the formation of cell aggregates, fomenting bacterial growth. Subsequent rounds of this cycle promote the formation of a stable granuloma in 3 days post-infection (*p.i*.), a process that is dependent on the region of difference-1 (RD1) virulence locus of *M. marinum* and independent of T-cells (Davis et al., 2002; Volkman et al., 2004, 2010; Davis and Ramakrishnan, 2009). It is unclear whether zebrafish Mϕs undergo polarization. Yet, since most transcription factors governing T-cell polarization are highly conserved in zebrafish (Mitra et al., 2010), along with physiological and pathological responses characteristic of type-1 and type-2 immunity (Aggad et al., 2010; Balla et al., 2010; Holt et al., 2011; Wittamer et al., 2011; Renshaw and Trede, 2012), it seems as a matter of time before Mϕ polarization is identified and characterized in this teleost. By contrast, the early stage of Mtb infection in mice is marked by M1 Mϕ polarization, reminiscent of clinical observations in TB patients (Benoit et al., 2008). In fact, transcriptomic analysis of infected murine Mϕs revealed the gene modulation provoked by Mtb overlaps with that of IFN-γ to establish the M1 program (Ehrt et al., 2001). Type-1 inflammatory signals secreted by infected Mϕs induce cell recruitment and formation of primary granulomas. Unlike zebrafish, however, granuloma formation in mice takes up to 3 weeks when *Mycobacterium* reaches a plateau and coincides with adaptive immunity involvement. For instance, nascent liver granulomas were visualized by intravital microscopy between 2 and 3 weeks after *Mycobacterium bovis* Calmette–Guerin (BCG) challenge (Egen et al., 2011). In another study, Mtb infection did not change the murine Mϕ population (iNOS^low^ARG1^low^) in bronchoalveolar lavage (BAL) during the first week (Redente et al., 2010). At day 21 *p.i*., however, M1 Mϕs (iNOS^high^ARG1^low^) dominated in BAL and granulomas, coinciding with a peak of IFN-γ in infected lungs (Redente et al., 2010). In humans, although NO production by monocyte-derived Mϕs remains controversial, both iNOS and NO are detected in granulomas and alleles for NOS2 are associated to TB susceptibility (Nicholson et al., 1996; Facchetti et al., 1999; Choi et al., 2002; Schon et al., 2004; Moller et al., 2009). After 35–60 days *p.i*., while murine Mϕs at the granuloma core remained iNOS^high^ARG1^low^, there was a dramatic shift toward the M2 program (iNOS^low^ARG1^high^) in Mϕs surrounding the core, accompanied by elevated type-2 inflammatory signals (Redente et al., 2010). This is in line with ARG1 detection in human TB granulomas (Pessanha et al., 2012).

**Figure 1:**
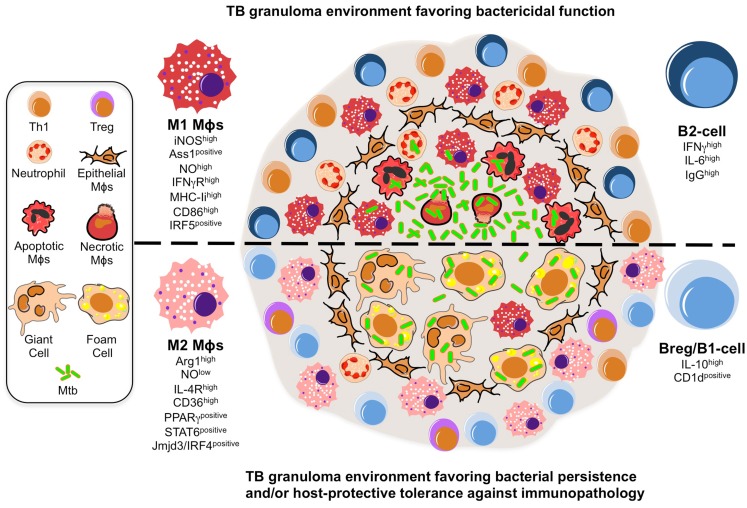
**A model illustrating the putative roles of Mϕ polarization and B-cell involvement during the formation and function of TB granulomas**. TB granuloma Mϕs undergo various specialized transformations: they can look like epithelial characterized by tightly interdigitated cell membranes that link adjacent cells; they can fuse into multinucleated giant cells; or they can differentiate into foamy cells with a high content of intracellular lipids. While none of these specialized transformations in the granuloma Mϕ population are well understood, we propose they might be reflection of the Mϕ polarization status that may render the granuloma structure with a microbicidal capacity (top) or as a tool of pathogenesis (bottom). In the former scenario, the local Mϕ population in lung undergoes a M1 polarization early on during Mtb infection and granuloma formation, distinguished by a cell-surface receptor repertoire responsive to pro-inflammatory signaling (e.g., IFN-γR^high^) and conducive for antigen-presentation (e.g., MHC-II^high^, CD86^high^), while acquiring a microbicidal capacity reflected in the NO production (e.g., *iNOS*^high^, ASS1^positive^), among others. These Mϕs have been noted to be most frequently located in the necrotic center of a mature tuberculous granuloma where apoptotic and necrotic Mϕs are abundant along with extracellular bacteria. Accompanying the M1 Mϕ polarization is the recruitment of neutrophils and Th1 cells, whose migration and activation status might be influenced by a B-cell involvement likely characterized by a pro-inflammatory phenotype (e.g., IFN-γ^high^ IL-6^high^ IgG^high^). In the latter scenario, we propose a change in the TB granuloma environment during the late stages of Mtb infection, distinguished by the M2 Mϕ polarization driven by the high expression of transcription factors (e.g., PPARγ^high^, STAT6^positive^) antagonistic for type-1 inflammation, and characterized by a cell-surface receptor repertoire promoting tissue repair activities (e.g., IL-4R^high^) and the formation of foamy cells (e.g., CD36^high^), while suppressing the microbicidal functions like NO production (e.g., ARG1^high^), among others. We envision M2 Mϕ polarization might give rise to the formation of foam and multinucleated giant cells, whose presence is noted to be most frequently at the rim and center of mature TB granulomas, and which may favor the intracellular resilience of Mtb. Furthermore, classical M2 Mϕs have been noted to be most frequently located surrounding the granuloma center and overwhelmingly in the local lung environment. Along with the M2 Mϕ polarization is the inhibition of neutrophil recruitment while enhancing that of T_regs_, activities that might be influenced by a B-cell involvement likely characterized by a anti-inflammatory phenotype (e.g., IL-10^high^, CD1d^positive^).

The shift toward M2 Mϕs during Mtb infection could have deleterious consequences for the granuloma as a host-protective structure (Figure 1). First, ARG1 expression in uninfected Mϕs surrounding the granuloma core suggests the development of an immunosuppressive niche. Indeed, Mtb promotes its survival by inducing ARG1 expression through MyD88-dependent signaling pathways (El Kasmi et al., 2008; Qualls et al., 2010). At the transcriptome level, murine M2 Mϕs displayed a diminished inflammatory response to Mtb as reflected by a reduced NO production and increased of iron availability, alluding ARG1 might also be implicated in nutrient deprivation mechanisms limiting microbial growth (Forbes and Gros, 2001; Kahnert et al., 2006; Cairo et al., 2011). Furthermore, M1 Mϕs possess a “fail-safe” system sustaining optimum NO production based on citrulline recycling via argininosuccinate synthase (ASS1), which is absent in M2 Mϕs (Qualls et al., 2012). Given the restrictive granuloma environment where arginine may be limited, the presence of this fail-safe system may become further accentuated. Second, M2 Mϕs may represent a transitional state into the formation of “foamy” Mϕs that are rich in cholesterol, a carbon source for microbial intracellular survival (Pandey and Sassetti, 2008; Peyron et al., 2008; Russell et al., 2009; Griffin et al., 2011). Recently, Mtb lipids were shown to trigger PPARγ, the master regulator of M2 polarization, to increase expression of CD36 and induce foam cell formation (Mahajan et al., 2012). Here, we postulate that factors governing M2 polarization establish additional anti-inflammatory signaling loops, like that of CD36, to increase microbial fitness within granulomas (Kuda et al., 2011). Third, the shift toward M2 Mϕs may allow Mtb to control the antigen-presentation process to undermine adaptive immunity within granulomas (Benoit et al., 2008). Indeed, TB granulomas display a limited antigen-presentation to evoke significant T-cell responses (Egen et al., 2011). While Mϕ polarization was not addressed in this study, M2 Mϕs do inhibit the proliferation of CD4 T-cells while fomenting the activity of regulatory T-cells (Schebesch et al., 1997; Curiel et al., 2004; Biswas and Mantovani, 2010). Altogether, the shift toward M2 Mϕs might also occur in human granulomas and contribute to Mtb pathogenesis given that TB susceptibility is often accompanied by elevated type-2 inflammatory and immunosuppressant signals (Kahnert et al., 2006; Raju et al., 2008; Almeida et al., 2009; Schreiber et al., 2009).

In the near future, we envision the role of Mϕ polarization in the granuloma context will be tested directly in different ways. First, we expect further advances in real-time imaging in both zebrafish and mouse models. Highly conserved Mϕ polarization markers are ideal candidates for the development of novel animal reporter lines expressing different fluorochromes to target the different Mϕ subsets. Second, specific gene inactivation of Mϕ polarization markers with the use of morpholinos (in zebrafish), siRNA-based technology, or gene-knockout strategy (including conditional strategies), may be used at different stages of granuloma formation in animal models. The strategies above could be used in combination with global array-based transcriptomics and proteomics approaches in order to assess the granuloma and local lung environment in the presence or absence of Mϕ subsets. Collectively, we expect there would be more future efforts to bridge results obtained in animals into the human context as discussed in the conclusion section.

## A Role for B-Cells in Granulomatous Diseases

Alterations in the lung environment by Mtb and/or subsequent immune responses likely affect the infection outcome. None of these is more apparent than the type-1 inflammatory storm that is unleashed in murine lungs at 3 week *p.i*., when a peak of IFN-γ/TNF coincides with CD4^+^ T-cell involvement, an event that impacts the organization of nascent granuloma structures. Yet, mice in which CD4^+^ T-cells are unable to produce IFN-γ/TNF are still resistant to TB, suggesting a complex scenario for protection (Torrado and Cooper, 2011). In this perspective article, we propose that, beside T-cells, B-cells modulate the TB granuloma formation and function through interaction with their cellular components.

Despite extensive evidence for anti-Mtb antibody production in TB patients (Kunnath-Velayudhan et al., 2010, 2012), and a higher susceptibility of pIgR (IgA receptor)-deficient mice (Tjarnlund et al., 2006), initial studies examining the role of antibodies in TB indicated a modest impact in protective immunity, with benefits limited to passive administration of anti-Mtb antibodies (Glatman-Freedman and Casadevall, 1998; Roy et al., 2005; Abebe and Bjune, 2009). This contributed to the notion B-cells played a minor role in TB immunity, if any. Yet, recent studies now provide compelling reasons to revisit the role of B-cells in TB (Cooper, 2009; Maglione and Chan, 2009; Flynn et al., 2011; Philips and Ernst, 2012). First, B-cells infiltrate the lungs of Mtb-infected mice and humans (Tsai et al., 2006), where they organize in ectopic B-cell follicles at the periphery of granulomas (Gonzalez-Juarrero et al., 2001; Ulrichs et al., 2004; Kahnert et al., 2007; Maglione et al., 2007). These foci are the predominant sites of cellular proliferation in the infected lungs attesting to the importance of B-cells in shaping the local environment during infection (Ulrichs et al., 2004). Moreover, B-cells also infiltrate the granuloma structure, as shown in non-human primates where activated B-cell clusters are found in close contact with T-cells (Phuah et al., 2012), and in the lungs from cattle with natural tuberculosis (Beytut, 2011). Mtb-specific B-cells also exist at local sites of infection in pleural fluids, a strategic place to influence the immunity against Mtb (Feng et al., 2011). Beyond TB, B-cells are well-known cellular components in several other granulomatous diseases (Table 1). Not only B-cells are present in granuloma but also they could be important for their maturation. This is suggested in pristane induced oil granuloma formation (Chen et al., 2010) and during *Schistosoma japonicum* infection (Ji et al., 2008) where the absence of B-cells results in a marked delay in granuloma formation. In the context of the TB, although granulomas form in the absence of B-cells, their numbers and size remain lower and they hardly become inflammatory (Bosio et al., 2000; Maglione et al., 2007). This could be the result of the well-known ability of B-cells to contribute to the organization of secondary and tertiary lymphoid organs (Moseman et al., 2012).

**Table 1:** **Characteristics of B-cells identified in non-TB granulomatous diseases**.

Disease or model	Type of B-cells	Reported role in disease	Specie	Reference
Wegener’s granuloma	Undefined	Detrimental	Humans	Voswinkel et al. (2008), Holle et al. (2012)
Sarcoidosis	Undefined	Unknown	Humans	Fukuda et al. (1997)
Churg–Strauss syndrome	Undefined	Detrimental	Humans	Donvik and Omdal (2011)
Crohn’s disease	B1^a^	Unknown	Humans	Geboes et al. (1986)
Schistosomiasis	Undefined	Favor protective Th2 immunity; inhibit T-cell-mediated immunopathology; granuloma formation	Mouse	Hernandez et al. (1997, Ferru), Jankovic et al. (1998), Jacobs et al. (1999), Ji et al. (2008)
Leishmaniasis	Include B2^b^ as well as CD5^+^CD1d^+^IL-10 producing regulatory Breg^c^ cells	Limits immunopathology; favor protective Th2 immunity; favor granuloma formation	Mouse	Smelt et al. (2000), Ronet et al. (2010), Moore et al. (2012)
Coccidioidomycosis	IL-10 producing B_regs_	Unknown	Humans	Li et al. (2005)
Paracoccidiois	B1, IL-10 producing B_regs_	Detrimental	Mouse	Popi et al. (2008)
Cat-scratch disease	IL-10 producing B_regs_	Unknown	Humans	Vermi et al. (2006)
Pristane induced oil granuloma response	Undefined	Granuloma formation	Mouse	Chen et al. (2010)

*^a^B1 cells: developmentally defined; innate-like B-cells in the mouse; CD5^+^ or CD5^−^ subpopulation poorly defined in humans*.

*^b^B2 cells: developmentally defined; include “conventional” follicular B-cells as well as “innate-like” marginal zone B-cells*.

*^c^B_regs_: functionally defined; present among various B-cell populations including CD5^+^CD1d^+^ B-cells; can produce IL-10*.

Second, although this is a rare event, occurrence of mycobacterial infections was reported upon rituximab-mediated depletion of B-cells, suggesting a protective role for these lymphocytes (Winthrop et al., 2008; Gea-Banacloche, 2010). However, other granulomatous diseases were successfully treated with rituximab (Donvik and Omdal, 2011; Holle et al., 2012), cautioning B-cells may be detrimental depending on the disease context. Finally, beyond antibody production, B-cells display diverse roles in the immunity against multiple pathogens that could operate during TB. In this regard, *Salmonella* infection, though not occasioning granuloma formation, represents a paradigm for antibody-independent roles of B-cells against an intracellular bacterium with the evidence that B-cells producing IL-10 (B_regs_) impairs the control of natural and vaccine-induced immunity to *Salmonella* (Neves et al., 2010). Since this role cannot simply be recapitulated in animal models lacking B-cells (Mastroeni et al., 2000; Mittrucker et al., 2000), this exemplifies how deletion of the B-cell compartment eclipses specific functions of these cells.

B-cells express adaptive and innate receptors to recognize pathogens (Blumenthal et al., 2009; Rawlings et al., 2012). Beyond antibody production, B-cells secrete various signals including cytokines, and serve as antigen-presenting cells (Rawlings et al., 2012). These immune-modulatory functions are performed by different B-cell subsets depending on their differentiation program (e.g., B1, B2), activation status (e.g., naïve, effector, memory), tissue distribution, the timing of the immune response, or disease context. From this perspective, the identity of B-cells infiltrating the lungs of TB patients or animals remains relatively unknown. In most cases, these cells (likely B2-cells) have undergone class switch recombination and produce antibodies (Phuah et al., 2012). However, CD5^+^CD1d^+^ B1-cells are also observed predominantly in TB patients (Zhang et al., 2012) and in mouse models of TB and other granulomatous diseases (Li et al., 2005; Popi et al., 2008; Ronet et al., 2010). Regardless of their identity or individual contribution, we estimate the B-cell compartment influences the TB granuloma formation and function through interaction with Mϕs, T-cells, and neutrophils (Figure 1).

As B-cells interact with Mϕs in TB granulomas (Tsai et al., 2006; Chakravarty et al., 2008), they might affect Mϕ polarization within these structures. A case in point, B1-cells differentiate M2 Mϕs via IL-10 *in vitro* and in a tumor model (Wong et al., 2010). However, mice deficient for B1-cells (xid model) displayed rather a susceptibility to mycobacterial infection, accompanied by increased levels of IL-10 (Junqueira-Kipnis et al., 2005; Russo and Mariano, 2010). Certainly, there are other B-cell subsets that could compensate as the *in vivo* source of IL-10, like B_regs_ (O’Garra et al., 1990; Lampropoulou et al., 2008). Likewise, there exist alternative *in vivo* immunosuppressive mechanisms driven by B-cells other than the B1-cell subset, as demonstrated for IgG production favoring FcR-mediated M2 Mϕ polarization in a carcinoma model (Andreu et al., 2010). In line with this observation, FCγRIIB-deficient Mϕs displayed a M1 Mϕ phenotype upon Mtb infection, express less IL-10 and better control the infection (Maglione et al., 2008). Since the phenotype manifests after 3 weeks of infection, IgG-producing B2-cells produced during the course of the adaptive immune response might be involved. B1 cells might rather contribute to M2 polarization through FcγR-independent IL-10-dependant mechanisms. Whether these events occur within the granuloma is currently unknown. Collectively, these studies infer a B-cell contribution to an immunosuppressive niche within TB granulomas by tilting Mϕs toward the M2 program.

If Mϕs are the main components in nascent TB granulomas, then CD4^+^ T-cells are perhaps the most critical component of stable TB granulomas as shown by the re-awakening of latent TB in HIV-1 co-infected patients. In recent years, multiple studies suggest an immune-modulatory role for B-cells in T-cell activity at the granuloma level. On one hand, B-cells can co-localize with T-cells in TB granulomas (Ulrichs et al., 2004; Beytut, 2011), and directly interact with them in the granulomas caused by *Leishmania* (Moore et al., 2012). On the other hand, B-cells influence T-cell effector functions either through cytokine production or antigen-presentation (Lund and Randall, 2010). In TB context, IL-10 derived from B1-cells controls the homeostasis of T-helper-17 (Th17), essential for anti-microbial immunity at epithelial/mucosal barriers (Zhang et al., 2012). Reciprocally, Th-17-associated cytokines promote the formation of B-cell foci in Mtb-infected mice, and correlate with B-cell infiltration in TB patients (Khader et al., 2011; Zhang et al., 2011). In the mouse model, IL-17A (Okamoto Yoshida et al., 2010) or IL-23-deficient (Khader et al., 2011; Zhang et al., 2011) animals have marked defects in the formation of granulomas and/or B-cell follicles. In addition IL-23-deficient mice also have poor levels of IL-17 and IL-22. These deficiencies resulted in a marked alteration of CXCL13 production, the chemokine responsible for B-cell recruitment and follicle formation (Khader et al., 2011; Zhang et al., 2011). It is not known if IL-10 production by B-cells is at the initiation or a secondary consequence of the alterations in IL-17 levels. These observations might provide an explanation for the links reported in TB patients between Th17 and formation of B-cell foci and IL-10 (Zhang et al., 2011, 2012).

Evidence obtained in non-TB diseases argue B-cells favor Th1 polarization (involved in TB protective immunity) through IL-6 and IFN-γ production during *Salmonella* infection, or promote Th2 differentiation (thought to be detrimental during TB) through either IL-2 (Wojciechowski et al., 2009) or IL-10 (Ferru et al., 1998; Popi et al., 2008; Ronet et al., 2010) in the control of different parasites. Conversely, B-cells also suppress T-cell activity as best illustrated in mice with a targeted deletion of MyD88 in B-cells during *Salmonella* infection (Neves et al., 2010). Finally, evidencing the role of B-cells as antigen-presenting cells, mice with a targeted deletion of MHC-II in B-cells displayed a reduction of IL-2 and IFN-γ by CD4^+^ memory T-cells during *Salmonella* challenge (Barr et al., 2010), and low pulmonary Th1 cell counts during *Pneumocystis* infection (Lund and Randall, 2010).

Another cell influencing TB granuloma formation is the neutrophil, whose migration can be controlled by B-cells. During *Salmonella* infection, mice with a targeted deletion of MyD88 in B-cells exhibited an accumulation of neutrophils in the spleen, an effect that likely depends on B_regs_-mediated IL-10 production (Neves et al., 2010). In the context of mycobacterial infections, aberrant neutrophil migration is known to have deleterious effects in host tissue integrity (Eruslanov et al., 2005; Berry et al., 2010). In mice deficient for the B-cell compartment (Maglione et al., 2007), Mtb infection leads to an uncontrolled accumulation of pulmonary neutrophils, an observation also supported by the excessive neutrophil migration in the peritoneum after BCG-vaccination (Kondratieva et al., 2010). These examples highlight the importance of tolerance mechanisms in TB.

Based on the above observation, it is tempting to propose that B-cell could act at different levels during TB such as during granuloma progression and by influencing the effector function of third-party cells like Mϕs. To directly examine this, studying B-cell contribution through comparison of B-cell-competent vs. B-cell-deficient animals should now be further complemented by studies examining the direct response of B-cells to Mtb infection, and through analyses in animal models lacking specific pathways in B-cells and biological consequences.

## Conclusion

Among trends emerging in TB etiology, the notion that the local lung environment shifts from a host-protective nature toward one favorable to microbial resilience is discussed here at the granuloma level and in the context of Mϕ polarization and B-cell function (see also an illustration in Figure 1). Exploring these issues will likely bring us closer to uncover the enigma concealed by TB granulomas. One can envisage that studies investigating the role of genes involved in host tolerance (Medzhitov et al., 2012) might be a good way to explore these aspects of the disease. Although in humans this could be limited to immunogenetic studies, more mechanistic studies could be conducted in animal models where selective inactivation of those genes could provide new insights on the consequences on the pathology. These studies could go along with more sophisticated approaches based on single cell analysis such as those involving laser microdissection or more global phenotypic signatures obtained from mass cytometry, in order to further identify cell subsets involved at different stages of granuloma formation and TB.

## Conflict of Interest Statement

The authors declare that the research was conducted in the absence of any commercial or financial relationships that could be construed as a potential conflict of interest.
